# Impacts of external shocks on the decisions of hog supply chains under liquidity constraints from the perspective of commercial credit

**DOI:** 10.1371/journal.pone.0212707

**Published:** 2019-03-04

**Authors:** Jiamei Wang, Xinhui Wang, Hongshu Li, Yuxuan Liu, Gangyi Wang

**Affiliations:** College of economics and management, Northeast Agricultural University, Harbin, Heilongjiang, China; The Bucharest University of Economic Studies, ROMANIA

## Abstract

Certain attributes of the hog industry increase the production risk in nodal enterprises of the hog supply chain, leading to high financing costs and eventually resulting in liquidity constraints. When the hog supply chain node enterprises are subjected to external shocks, on the basis of the commercial credit relationship in the supply chain, the entire supply chain generates liquidity risks and systemic risks. We analyze the input and output of the hog supply chain node enterprises under the constraint of liquidity, construct the mathematical model, discuss the dynamic differences of liquidity constraints in different situations, and measures the commercial credit risk and anti-risk ability of the pig supply chain node enterprises. If the external shock is less than a certain value, the current profits of the hog enterprise can entirely make up for the loss caused by external shocks, and the production of the firm will return to its state of equilibrium. If the external shock is large enough, liquidity constraints will seriously restrict the production input of the enterprise, which then leads to a deceleration of production input and may ultimately result in bankruptcy. We believe that the structure of the hog industry supply chain should be constantly adjusted to optimize the industrial upgrading and organizational form of the hog supply chain.

## Introduction

The hog industry has been an important part of China's agricultural sector; the consumption of pork accounts for over 60% of China’s total meat consumption. The hog industry features a few dominant large enterprises and most are small and medium-sized enterprises, the most representative of which are hog-breeding companies. The main body of the culture is still dominated by retail investors, accounting for up to 70%. As a result of asymmetric information and agency costs, investment in and financing of hog-breeding enterprises often face severe liquidity constraints. Traditional lending cannot fully meet the capital needs of industrialized agriculture, and small-scale private enterprises face serious credit rationing and credit discrimination in the present bank credit system [1]; thus, liquidity constraints are still a serious problem faced by hog enterprises.

With the gradual entrance of informal financing into the financial market, commercial credit has arisen as a type of alternative financing vehicle to fill the financing gap for these enterprises, and it has effectively alleviated their liquidity constraints [2]. Cunat V [3] found that small enterprises find it difficult to engage in the capital markets because of credit rationing, credit constraints and other factors; however, suppliers have comparative advantages in corporate information, they are willing to provide funds to enterprises. Commercial credit between an enterprise and its suppliers widens the enterprise's financing channel and alleviates liquidity constraints [4] Allen *et al* [5], Duan, Yang and Zhang [6], Sun *et al* [7] have found that commercial credit is useful for enterprises—particularly small and medium sized enterprises, private enterprises—that are facing difficult external financing problems; in some instances, commercial credit may even exceed bank credit and can effectively alleviate liquidity constraints. These scholars have conducted substantial research regarding the costs of and reasons for commercial credit financing and have demonstrated that commercial credit can alleviate liquidity constraints; however, they have not conducted a deep analysis of how enterprises, especially hog enterprises, obtain financing through commercial credit to alleviate such liquidity constraints. Upstream and downstream nodal enterprises in the hog supply chain are bound to one another through money and/or the supply of goods using commercial credit. However, a rupture in the supply of capital could trigger a single enterprise’s capital risk and even systemic risk across the entire supply chain. Supply chain risk is an uncertainty factor (such as the occurrence of accidents) that injures one or more members in the chain but damages the operation of the entire supply chain, such that the operation cannot meet its goals [8]. In fact, when unpredictable external shocks enter into the operational process of an enterprise, such as those affecting the entire capital market, macro-economic uncertainty is exacerbated, which can lead to rapid declines in output, employment and productivity [9]. By understanding the causes of external shocks and identifying the external shocks that may occur, the unpredictability of external shocks can be decreased, which can allow hog enterprises to address the causes in advance [10]. Summarized those factors related to nodal enterprises suffering from liquidity constraints in the hog supply chain: the number of sows and others (including fodder prices), on the one hand, and external shocks (including the GDP growth rate and serious diseases, among others) on the other hand. There are different forms of external shocks, and their impacts on the hog supply chain are also different. In addition, the measures of nodal enterprises will change when they face different liquidity constraints caused by different forms of external shocks. Thus, external shocks not only influence the production of individual hog enterprises but also the stability of the whole supply chain. Some scholars used the impulse response function to analyze the impact of external shock on the price transmission mechanism of the pig industry chain, and found that the impact of external shock on the price fluctuation of pigs reached more than 90% [11].Based on the research content of the above scholars, we found that the point at which external shocks enter into nodal enterprises in the hog supply chain will affect the decisions and behaviors of the enterprises. Due to the requirement of liquidity, enterprises in the hog supply chain will engage in input and output adaptive adjustments; hog-breeding enterprises and slaughter enterprises will cooperate with one another in the form of credit or prepaid commercial credit. This article will mainly explore the following questions. How do external shocks restrain hog enterprises’ liquidity when operating in the context of commercial credit? Based on commercial credit, when external shocks enter into the hog supply chain, will the shock spread to upstream and downstream enterprises, and how much liquidity risk will it cause? What systemic risks will external shocks cause regarding the operation of the entire supply chain? By analyzing the above problems, we seek to reveal the threshold value of the external shocks that nodal enterprises in the hog supply chain might face. Based on these thresholds, we can assess when nodal enterprises are facing different levels of external shocks and set forth appropriate and reasonable suggestions based on these results.

## Materials and methods

### Structure of the hog supply chain

According to the different core enterprises, the current organizational structure of the hog supply chain in China is divided into a vertical integration structure and a strategic alliance structure.

#### Vertical integration structure

**Fig 1:**
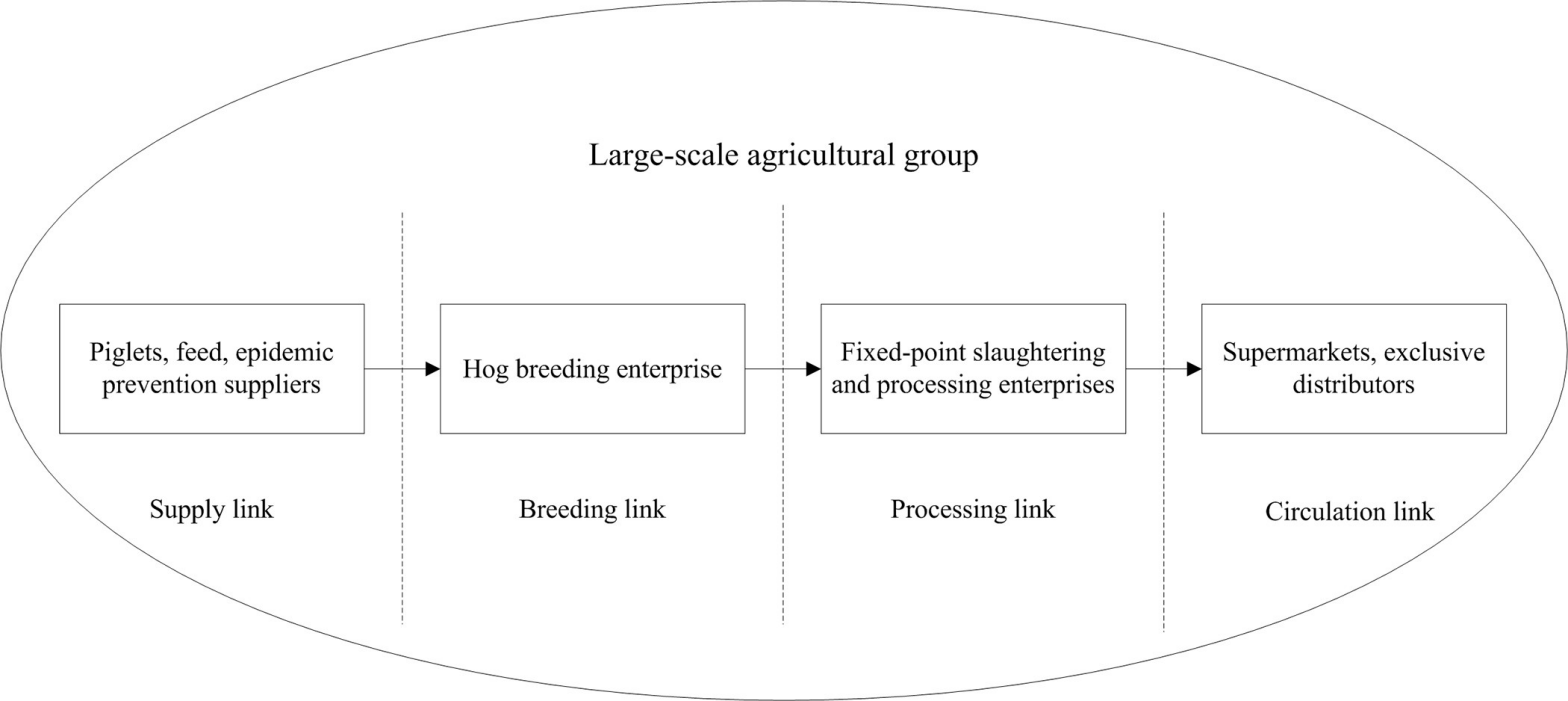
Vertical integration structure.

Vertical integration indicates that a large agricultural group extends its industrial structure both upstream and downstream simultaneously, which includes the business functions for all links and nodes in the entire supply chain. Among them, the link refers to supply, breeding, processing, and circulation links, the node refers to suppliers, hog breeding enterprise supermarkets, etc. At present, some large enterprises have adopted this organizational structure, in which the supply chain is both vertical and integrative, thus allowing these enterprises to simultaneously practice green management and maximize profits **Fig 1**.

#### Strategic alliance structure

**Fig 2:**
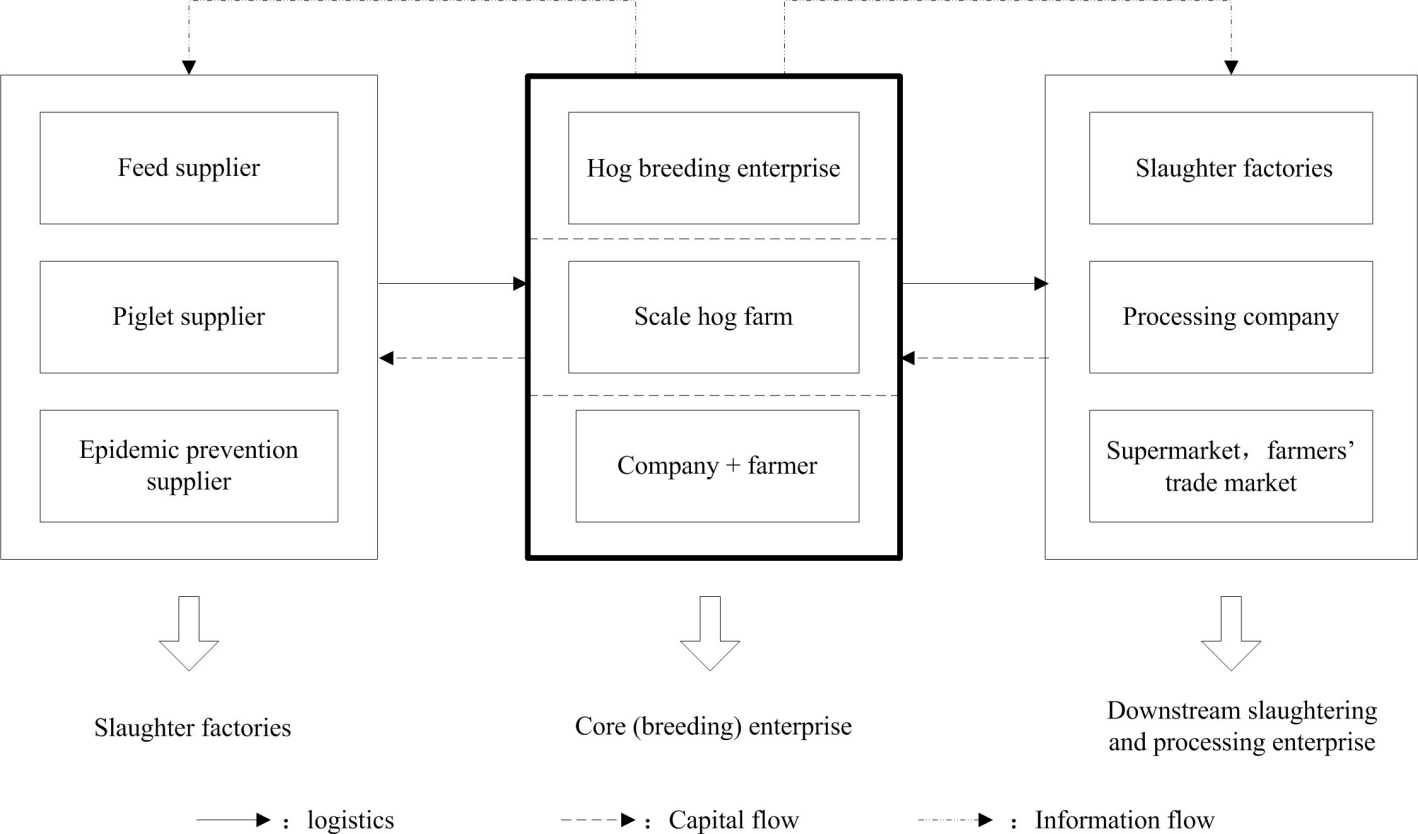
Strategic alliance structure.

The strategic alliance structure is divided into three segments. Specifically, the hog breeding business is a core enterprise, slaughter processors are a core enterprise, and the large retailers are a core enterprise. We use the hog breeding leading structure as an example to analyze liquidity constraints **Fig 2**. The hog breeding leading structure is defined by a large-scale hog breeding enterprise or farm as the core of the supply chain, and the hog breeding enterprise purchases raw materials from upstream suppliers and then raises healthy hogs to meet the order demand of its downstream partners. Meanwhile, the enterprise performs well in the market due to is core strength, and technical information is transmitted to both upstream and downstream enterprises, thus promoting information sharing and financial flows throughout the entire chain. The supply chain structure distinguished by property rights has a different capital allocation function that addresses different-sized liquidity constraints. A strategic alliance structure with producers, slaughter processors or large retailers as the dominant enterprise faces different liquidity risks and system risks due to the different types and scales of external shocks.

### Method selection

When external shocks occur, liquidity will lead to more serious restrictions in the fixed investment and capital spending activities of the enterprise; moreover, the more external the accident caused by external shocks, the tighter the company's liquidity will be and liquidity constraints will be more serious, such that the enterprise’s demand for money will be felt more strongly [12]. For the hog supply chain, external shocks can result from natural risks, such as epidemics, rainstorm debris and market risk (such as hog market price abnormal fluctuations).

In 1997, Nobuhiro Kiyotaki and John Moore conducted a theoretical study on the liquidity constraints that external shocks propagate through supply networks to companies that borrow from each other [13]. In 2012, Crescenzo dell 'aquila and Mario Eboli proposed a trade credit chain model [14] based on Nobuhiro Kiyotaki and John Moore's trade credit chain model, and used this model to measure the risk degree of such investment decisions on liquidity risk and unexpected liquidity restriction caused by systemic risk. By describing the threshold value of external shocks, this study analyzed the various impacts of liquidity shortage caused by external shocks on enterprises' investment and output in the agricultural food supply chain, aiming to evaluate the role of trade credit in improving enterprises' ability to resist liquidity shocks.

Inspired by the work of Kiyotaki and Moore, this paper draws on the models used in the study of the liquidity constraints of agricultural product supply chains by Crescenzo dell’Aquila and Mario Eboli. The fixed proportion set in the original model supply chain equilibrium state is changed to the ratio of labor and raw materials. This model is mainly composed of two groups of media: Banks and enterprises. Enterprises can be buyers or suppliers in the industrial chain, or buyers and suppliers in a certain link. In addition, there are two types of companies: ordinary companies with liquidity restrictions due to credit rationing, and strong companies with no liquidity restrictions or access to external or internal financing to alleviate temporary liquidity shortages. The model focuses on production capital, and describes the production function as a function of working capital investment only, which is applicable to stable production level in equilibrium state. The model assumes that the time is divided according to the cycle, and the cycle length corresponds to the production cycle. Suppose the supplier never asks to liquidate the defaulting buyer and always accepts to defer payment until the next cycle; Assume that the production function has a constant return on scale and that the combination of the variables of production (labor and inventory) is fixed.

This study focuses on hog supply chains, including hog breeding enterprises, slaughter factories, retailers, and other nodes of varying strengths. Because of pig breeding is an indispensable core-sector in the hog supply chain, we set the hog breeding company as the core enterprise for research in the following text. By building an input and output mathematical model of commercial credit, quantifying the external shocks and their losses, the fund flow equation under equilibrium state is established. In the non-equilibrium state model under external impact, the liquidity risk suffered by the hog breeding enterprises is judged by classifying the external impact size, and then the systemic risk of commercial credit formation is evaluated.

In case analysis in this article, we use the cost-benefit data [15] of Chinese hog breeding industry to determine the size of mathematical model parameters, and introduce into the input and output model of the hog breeding enterprise under equilibrium. By deducing and calculating the critical value of external shocks, namely liquidity constraint and risk threshold, the liquidity risk of hog breeding enterprises is evaluated. Furthermore, the model is expanded to measure the entire supply chain output gap of the hog breeding enterprise, and then measure the systemic risk of commercial credit formation.

### Model assumptions

By reviewing the related research literature, we found that the working capital of nodes in the hog supply chain mainly come from internal and external financing [16]. Among these, external financing mainly includes bank loans and commercial credit. Therefore, this article stipulates that external funding used in the production of enterprises mainly comes from upstream suppliers and banks extending commercial credit between one another and that the priority of enterprises is to repay bank loans. Based on the mechanism of commercial credit, when a hog-breeding company does not pay off all the payments owed by the end of the current period, the supplier does not require liquidation of the defaulting company’s assets and accepts deferred payment into the next cycle. Based on the above analysis, the model has the following assumptions and limitations:

#### Cycle assumptions

(1) The time dimension is divided into several periods (written as T) and is concretely expressed as the hog breeding period (generally 4 ~ 5 months) [17]; (2) At the end of each production period, the current inventory of hogs that have remained will be retained until the next period; (3) The hogs bred in the previous period are sold in the current period and are thus used for payback at the end (credit sale), and the repayment period of the commercial credit is equal to the hog breeding period.

#### Parameter setting

This study establishes the following parameters [14]: *I* represent investment of hog-breeding enterprises in raw materials; *L* is indicating labor; *w* denotes remuneration for workers; *r* is bank lending rates; *δ* is commercial credit interest rates (*δ*>*r*); *σ* denotes exogenous shocks (quantified as the amount of loss generated by shocks); *λ* expresses liquidity shortage; and *α* denotes input and output elasticity.

### Model construction

#### The equilibrium state

Assume that N is a set consisting of n firms. Each firm links upstream and downstream using commercial credit. We take one hog breeding company as the core firm, denoted as *a*, its supplier as *b*, and its buyer as *c*. The equilibrium state of the supply chain means that the operating capital for production is adequate and that output is optimal. We assume that the optimal amount of output is y* and that the price of output is equal to unity. Accordingly, the assumptions regarding the equilibrium state of the supply chain are as follows:

1. The labor which means the number of workers in company *a* and inventory ratio is fixed as: LI=k;
2. The output in production is expressed by yield as, and the yield is linear to the current investment:
  y=α(I+wL)=Iα(1+kw)

At the beginning of the period, firm *a* buys inventory from *b* for production purposes. At the end of the period, the earnings are used to pay the bank loan and interest. Firm *a* then pays the supplier for the goods and retains any surplus as profit. The specific functions of the variables and the flow chart are expressed as follows. Firm *a* buys inventory I* from firm *b* to produce in the current period, which is expressed as y* = I*α(1+*kw*). Firm *a* receives sales revenue, y*(1+*δ*), at the end of the period. This revenue is used to pay off bank loans (simplified as B) and interest for *B*(1+*r*) and goods to *b* for I*(1+*δ*), and the rest represents corporate profits in the equilibrium state, expressed as π*. The capital flow equation for the equilibrium state is:

$$y^{*}(1+\delta )=B(1+r)+I^{*}(1+\delta )+\pi^{*}$$

#### Non-equilibrium state under external shocks

Some external shocks that occurred in the T period of hog breeding destroy the original equilibrium state. The loss generated by the external shocks is undertaken by profits π* on sight. We assume that the loss is so enormous as to cause firm *a* to default; thus, *σ*>π*. In this case, the sales revenue generated at the beginning of the period cannot pay, absolutely, the operating costs:

$$y^{*}(1+\delta )<B(1+r)+I^{*}(1+\delta )+\sigma$$

Because hog breeding firm *a* cannot repay the accounts payable to its supplier *b*, the liquidity shortage equals *λ* and is generated *λ* = *σ*-π*. The impact of the liquidity shortage on the enterprise's investment occurs in the T+1 period. According to the assumption, supplier *b* does not ask firm *a* (which is in default) to liquidate property, and it accepts *a* delay in payment into the next period. Firm *a* must repay debts *λ*(1+*δ*) generated by the delay in payment at the end of period T+1, which may lead firm *a* to not reach its optimal output y* due to *a* lack of capital. At the beginning of the T+1 period, *b* is willing to continue to grant commercial credit if *a* is able to repay all debts. The capacity of *a* to repay debts to *b* depends on the size of the external shocks and corporate profits. When $\sigma <\pi^{*}(1+\frac{1}{1+\delta })$, firm *a* returns to equilibrium production status by the cycle of surplus. When facing maximum investments, the earnings of *a* in the T+1 period cannot completely make up for the liquidity shortage. Firm *b* grants a commercial credit when it grasps the true situation of *a*, and the credit volume is less than repaying the capability of *a*. Therefore, the input of inventory *I*_*T*+1_ must be less than the theoretical level, I*. The loss caused by the external shocks affects the production of firm *a* in the T+2 period. In the T+2 period, the decrease in production in the T+1 period brings about a proportional decrease of earnings in the T+2 period. If:

$$y_{T+1}(1+\delta )<B(1+r)+I^{*}(1+\delta )$$

Then, the inventory input in the T+2 period remains limited by the liquidity shortage. By I*α(1+*kw*) = y*, we can attain:

$$\left[y^{*}-\left(\alpha \left(1+kw\right)(\lambda -\frac{\pi^{*}}{1+\delta })\right)\right]\left(1+\delta \right)<B\left(1+r\right)+y^{*}\left(\frac{1}{\alpha (1+kw)}\right)\left(1+\delta \right)$$

The production of firm *a* in the T+2 period is still affected by external shocks if inequality was established. In contrast, the earnings generated in the T+1 period can recover production to the state of equilibrium in the T+2 period. By *λ* = *σ*-π*:

$$\sigma >\pi^{*}\left[1+\left(\frac{1}{1+\delta }+\frac{1}{\left(1+\delta )\alpha (1+kw\right)}\right)\right]$$

The investment in the T+2 period will then be reduced due to the liquidity shortage, which is expressed as *I*_*T*+2_<I*; the computing method of the reduction of the input and output in the T+2 period is the same as that in the T+1 period; the input and output in the T+2 period further declines, *I*_*T*+2_<*I*_*T*+1_; by *λ* = *σ*−π*:

$$\sigma >\pi^{*}\left[1+\frac{\alpha (1+kw)}{\left[\alpha (1+kw)-1\right](1+\delta )}\right]$$

In the T+2 period, the output of the firm reduces continually as a result of the external shocks, but the firm may recover production to the equilibrium state by internal financing if the loss due to external shocks is less than a certain amount. In the production periods following T+2, available liquid capital is generated by production surplus in the previous period. We assume that returns to scale remain the same and that the capital used for internal financing is accumulated gradually starting during the T+2 period. The production of the firm then recovers, step-by-step, to a state of equilibrium.

According to the above deduction, the sizes of the external shocks suffered by the firm are classified as follows:

$$\sigma_{1}=\pi^{*}(1+\frac{1}{1+\delta })$$ (1)

$$\sigma_{2}=\pi^{*}\left[1+\left(\frac{1}{1+\delta }+\frac{1}{\left(1+\delta )\alpha (1+kw\right)}\right)\right]$$ (2)

$$\sigma_{3}=\pi^{*}\left[1+\frac{\alpha (1+kw)}{\left[\alpha (1+kw)-1\right](1+\delta )}\right]$$ (3)

According to Eq (1), when 0≤*σ*<*σ*_1_, the current yield can recover the loss from external shocks and will not affect the stable production in the next period as a result. According to Eq (2), when *σ*_1_≤*σ*<*σ*_2_, the sales revenue in the current period cannot recover the liquidity shortage, which makes the input and output in the T+1 period restricted: *I*_*T*+1_<I* and *y*_*T*+1_<y*, namely, the input and output in the T+1 period are both less than in the equilibrium state. According to Eq (3), when *σ*_2_≤*σ*<*σ*_3_, liquidity constraints caused by external shocks cannot be made up in the T+1 period and stretch to the T+2 period, which makes the input and output in the T+2 period restricted and less than they were in the equilibrium state: *I*_*T*+2_<I* and *y*_*T*+2_<y*; when *σ*>*σ*_3_, the liquidity constraints caused by external shocks are strong enough to slow down the input of raw materials by the enterprises and make the input and output in the T+2 period less than they were in the T+1 period, namely *I*_*T*+2_<*I*_*T*+1_ and *y*_*T*+2_<*y*_*T*+1_. When $\sigma >\pi^{*}(1+\frac{1}{1+\delta })$, external shocks not only will affect the production of the firm but also can provide risk conduction to both upstream and downstream enterprises by means of commercial credit, resulting in a decline in output over the entire supply chain.

### Model extension

The modeling process above meets the numerical analysis of core enterprises in the hog supply chain, it is also applicable to the assignment of the entire chain characteristic parameters including industrial and trading enterprises and the calculation of liquidity risk. The external shocks suffered by one firm in the hog supply chain are transmitted to the other firms, thus generating systemic risk due to the combination of upstream and downstream firms using commercial credit. By further expanding the liquidity risk measurement model, we calculate the output gap of the entire supply chain to measure the systemic risk generated by commercial credit.

To ensure the integrity and objectivity of model inference, Based on the model of this paper, the following points are proposed. (take an example of three node enterprise *a*, *b*, *c* in the supply chain upstream and downstream):

1. The loss of external shocks suffered by firm *a* in the supply chain is equal to *σ* and *σ*>π*. The liquidity shortage resulting from external shocks is recorded as *λ*_a_ = *σ*−π*.
2. Supplier *b* allows firm *a* to repay debt by deferring it to the next period. The deferred revenue from the liquidity shortage of firm *b* is now *λ*_*b*_, and *λ*_*b*_ = *λ*_*a*_−π* = *σ*−2π*.
3. If firm *b* is a strong firm with substantial capital, it will recover the liquidity shortage by way of a bank loan or liquid reserves and thereby inject liquidity into the supply chain to stop the systemic risk.
4. If firm *b* is unable to obtain liquidity, it must break the contract with supplier *c*.
5. The systemic risk ends when firm *c* is a strong firm; otherwise, the liquidity shortage is transmitted to downstream firms, which, in turn, leads to liquidity shortage *λ*_*c*_ = *λ*_*b*_−π* = *σ*−3π*. A liquidity shortage can be recovered by the retained profits of firms that are affected by external shocks.
6. We assume that the profits π* of all firms in the equilibrium state are equal in N-sets. As long as there is no strong firm, the number of companies affected by the system risk is greater than or equal to the minimum value of natural numbers σπ*. Setting all firms involved in default resulting from the external shocks of firm *a* to D and every subset as m, we then number them as j = 1,2,3,…m. (j-1) is the number of firms from *a* to *j* in the transmission chain, the default money of firm *j* suffered from its downstream firm is *σ*−(*j*−1)π*, and the liquidity shortage of firm *j* is *λ*_*j*_ = *σ*−jπ*.

We can analyze the impact of liquidity risk on the above firm’s input and output; then, we can calculate the yield gap of the supply chain.

According to the above, the yield gap of firm *a* in the period when it suffered external shocks is equal to: $y^{*}-y_{T+1}=\alpha (1+kw)(\lambda_{j}-\frac{\pi^{*}}{1+\delta })$. We assume that there is no strong firm in set D; therefore, the yield gap of the entire supply chain in the T+1 period is:

$$m\left(\sigma -\left(\frac{\pi^{*}}{1+\delta }\right)\right)\alpha (1+kw)-\sum \begin{array}{c} m\\ j=1 \end{array}j\pi^{*}\alpha (1+kw)$$ (4)

In contrast, if firm *j*(*j*<*m*) is the strong firm in the supply chain, the yield gap is equal to:

$$\sum \begin{array}{c} j-1\\ j=1 \end{array}\left(\sigma -j\pi^{*}-(\frac{\pi^{*}}{1+\delta })\right)\alpha (1+kw)$$ (5)

If the external shock that firm *j* suffers is *σ*>*σ*_2_, the yield gap in the T+2 period is equal to: $y^{*}-y_{T+2}=\alpha (1+kw)\left[\alpha (1+kw)\left(\lambda -\frac{\pi^{*}}{1+\delta }\right)-\frac{\pi^{*}}{1+\delta }\right]$. The yield gap of the entire supply chain in the T+2 period is:

$$\sum \begin{array}{c} m\\ j=1 \end{array}\alpha (1+kw)\left[\left(\sigma -\left(j+1\right)\left(\frac{\pi^{*}}{1+\delta }\right)\right)\alpha \left(1+kw\right)-\left(\frac{\pi^{*}}{1+\delta }\right)\right]$$ (6)

If firm *j*(*j*<*m*) is the strong firm in the supply chain, the yield gap is equal to:

$$\sum \begin{array}{c} j-1\\ j=1 \end{array}\alpha \left(1+kw\right)\left[\left(\sigma -\left(j+1\right)\left(\frac{\pi^{*}}{1+\delta }\right)\right)\alpha \left(1+kw\right)-\left(\frac{\pi^{*}}{1+\delta }\right)\right]$$ (7)

### Calculation example

**Fig 3:**
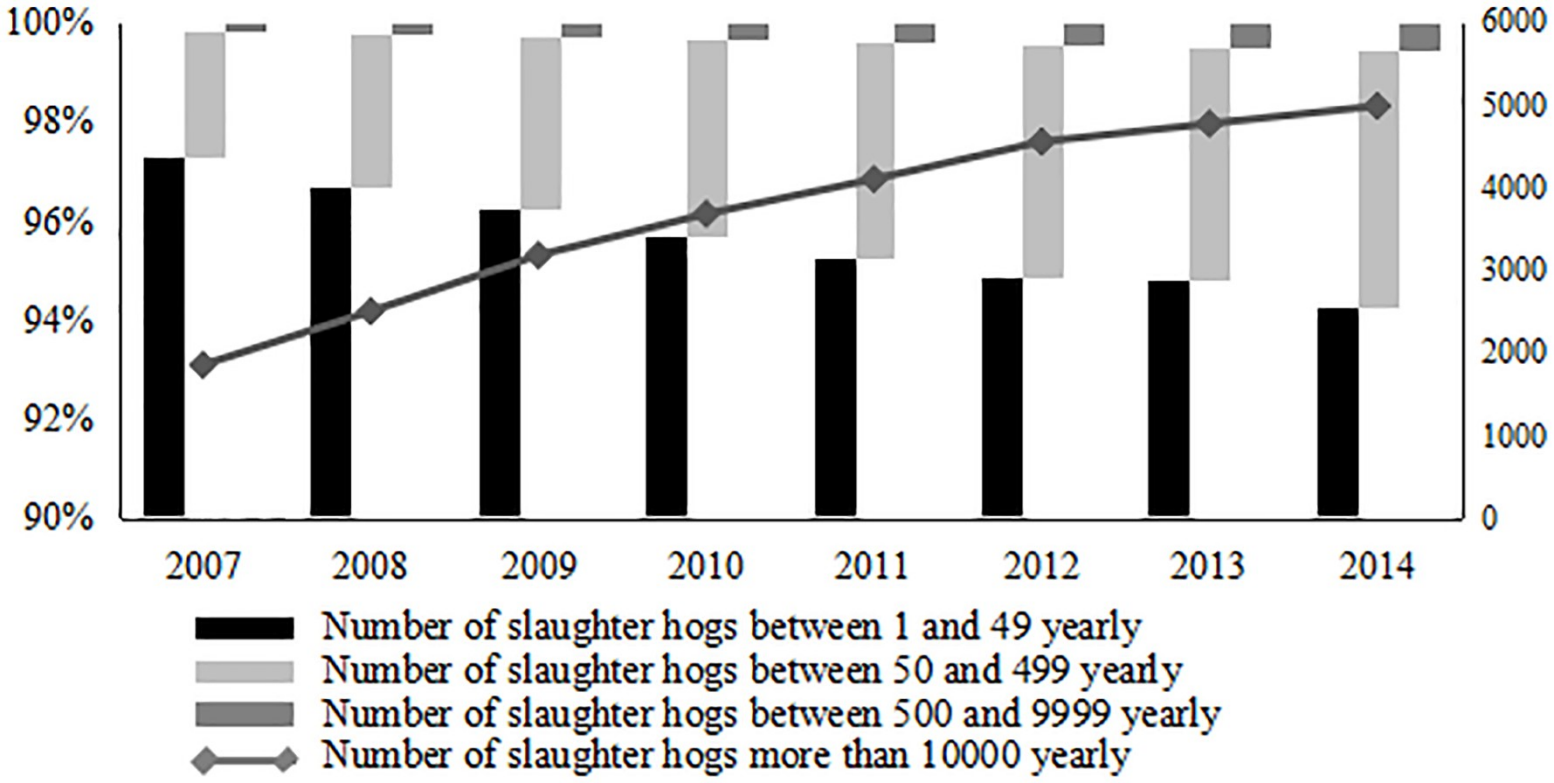
Trend of hog breeding scale in 2007–2014.

The characteristics of the hog supply chain determine the type and size of external shocks that the chain can withstand. Considering the differences in the productive efficiency of different hog breeding scales, according to the standard of classification of the *National Agricultural Product Cost and Benefit Data Compilation* [15], the scales of hog breeding were divided into four types: retail, small-scale, medium-scale and large-scale (details in **Table 1**).According to data from 2007 to 2014 in *CHINA ANIMAL HUSBANDRY AND VETERINARY YEARBOOK* [17], as the number of years increases, the proportion of retail investors decreases Fig 3. Retail investors gradually withdrew from the market with the evolution of large-scale farming and hog industrialization. and production in the supply chain prioritized large-scale farming.

**Table 1:** Annual slaughter of hog of four kinds of scale.

Breeding scale	Standard of Classification Quantity(Q)			
	Retail	Small-scale	Medium-scale	Large-scale
Annual slaughter	Q≤30	30<Q≤100	100<Q≤1000	Q>1000

We take the medium-scale farming firms as an example, According to the data in *National Agricultural Product Cost and Benefit Data Compilation* [15], the average number of average feeding days, labor cost, feed cost, and net profit per unit of hog are calculated as the data used in this paper from 2007 to 2014, as shown in **Table A in S2 File**. According to Table 1, medium-scale hog breeding enterprises produce 100–1000 hogs per year. Therefore, a median of 550 is selected as the annual production of medium-scale hog breeding enterprises in this example. Among them the production period of medium-scale hog farming **T** is approximately 144 days, the corporate profit π* for each period is approximately 45,600 yuan(Enterprise profit = Profit*Average feeding days /365*550), the labor wage *w* for each period is approximately 22,700 yuan(Workers' remunerration = Labor cost*Average feeding days/365*550), and *k* is 0.13(*k* = Labor cost/Feed cost), which is determined by calculating the ratio of labor costs and feeding costs for each unit of hog.

The firms in the hog supply chain obtain short-term liquidity loans in the form of guarantees for one another. The commercial loan interest rate of financial institutions has a certain degree of rise on the basis of the benchmark interest rate. In 2013, the People's Bank of China fully liberalized the loan interest rate control, and the lower limit of loan interest rate was removed, which meant that the loan interest rate was liberalized. Take the proportion of each loan interest rate interval in 2013 as an example. According to the **Table B in S2 File** of the ratio of interest rates of RMB loans of financial institutions in 2013 in *Almanac of China's Finance and Banking* [18],the proportion rising by 1.1~1.3 times on the basis of the benchmark interest rate was the largest. In 2013, the rising range of this interest rate accounted for 25.7% on average, and 1.2 times of the average was selected as the loan interest rate of breeding enterprises in formal financial institutions. The interest rate of commercial credit loan shall be specifically set by the enterprise providing the loan. But according to article 6 of the *Several Opinions of the Supreme People's Court on the People's Courts' Trial of Lending Cases*, the interest rate of private lending may be appropriately higher than the interest rate of the bank, and the people's courts in various regions may have specific control according to the actual situation of the region, but the highest shall not exceed four times of the interest rate of similar loans of the bank (including the interest rate principal). Beyond this limit, the excess interest shall not be protected. Through investigation, we found that the interest rate of commercial credit loan was rarely more than twice that of formal financial institutions, so we select twice the interest rate of formal loan as the interest rate of commercial credit.

As shown in **Table C in S2 File [18]**, the benchmark interest rate for RMB loans of financial institutions during in the 8 years is 6.39%, so the commercial credit interest rate is 15.34% (Commercial credit interest rate = Benchmark interest rate*1.2*2).

And the input and output elasticity *α* of scale farming is approximately 0.84 [19], The sorted parameter comparison table is shown in **Table 2**.

**Table 2:** Parameter comparison.

Parameter	π*	*w*	*k*	*δ*	*α*
**Meaning**	enterprise profit	laborer's salary	technical coefficient	commercial credit interest	input-output elasticity
**Value**	4.56	2.27	0.13	0.1534	0.84

Commercial credit rates shall be formulated by the specific firm. We assume that the formal loan interest rate of the hog firm is 1.4 times the benchmark interest rate and that the commercial credit rate is 2 times that of the formal loan interest rate.

## Results

Inputting the values above into formulas (1), (2), and (3) yields the following results: *σ*_1_ = 8.51, *σ*_2_ = 12.15, *σ*_3_ = 53.5 (the unit is ten thousand yuan). According to these results, we can arrive at the following basic results:

Result 1: For a middle-sized hog farming firm, if the external shock is in the range of 0~8.15, current earnings can compensate for the loss due to external shocks and will not affect the stable production of the firm in the next period for a medium-scale hog breeding firm.
Result 2: If the external shock is in the range of 8.15~12.15, current earnings cannot completely recover the liquidity shortage, which makes the input and output in the T+1 period lower than the input and output obtained in a state of equilibrium.
Result 3: If the external shock is in the range of 12.15~53.5, the sales revenue in the T+1 period cannot completely recover the liquidity shortage, and the input and output in the T+2 period will remain limited and will be lower than the input and output obtained in a state of equilibrium.
Result 4: If the external shock is greater than 53.5, it will lead to a reduction in inventory investment for the middle-sized hog farming firm, namely the input and output in the T+2 period will be lower than the input and output obtained in the T+1 period, which might cause it to go bankrupt.

In order to calculate the output gap of the entire supply chain to measure the systemic risk generated by commercial credit. In order to further measure the entire supply chain output gap of medium-scale hog breeding enterprises, and then measure the systemic risk of commercial credit formation, according to the above formula and related parameters (4), (5), (6), (7), For example, we assume that there are ten firms involved in the default in the hog supply chain. which indicates that m = 10. Among the enterprises related to hog breeding, including feed farming, piglet breeding, slaughtering and processing, disease control, fertilizer, professional equipment purchase, maintenance and sales, etc., there are mainly about 10 enterprises in the entire industrial chain as a common phenomenon, so choose m equals 10; Among them, strong and powerful companies generally refer to enterprises that have an active position in the pricing of capital resources and even pig products in enterprises such as feed, equipment, slaughtering and processing. And if we assume that j = 5, we can obtain the following basic results:

Result 5: When *σ*_1_≤*σ*<*σ*_2_, if there is no strong firm in the supply chain, the yield gap of the entire supply chain is 10.88 *σ*-315.85 by taking the numerical into type (4). If the fifth firm is a strong firm, the yield gap of the entire supply chain is 4.35 *σ*-66.81 by taking the numerical into type (5).
Result 6: When *σ*_1_≤*σ*<*σ*_2_, if there is no strong firm in the supply chain, the yield gap of the entire supply chain is 11.83 *σ*-347.14 by taking the numerical into type (6). If the fifth firm is a strong firm, the yield gap of the entire supply chain is 4.73 *σ*-82.71 by taking the numerical into type (7).

We can determine the fiscal subsidization of the development of the hog industry and the direction and amount of the financial support based on the exact value of *σ*, which yields a scientific basis for financial services for agricultural financial institution innovation and solves the obstruction and optimization of the cash flow in the hog supply chain and optimizes the liquidity of the working capital of hog enterprises and the capital supply chain. The measuring methods and results in this section are practical. If combined with real-time data from the hog supply chain, related enterprises in the chain can gain timely access to finance, the financing risk can simultaneously be transferred to the strong member in the supply chain, to realize the close integration between the hog industry supply chain and the capital supply chain.

## Discussion

With serious information asymmetry and credit rationing in the capital market, liquidity constraints inevitably appear in firms of different sizes. Furthermore, nature restricts the production operations of firms and thus affects the strategic decisions regarding investment and financing. The liquidity constraints hypothesis considers that the investment level of a company depends on the level of profit or anticipated profit as a result of imperfect capital markets. Commercial credit has increased in the financial market due to its financial function, the stabilizing order, the quality of inventory and other advantages; it effectively eases liquidity constraints for a company as a type of alternative financing [2–4]. Recent data indicate that the cost of hog breeding is becoming increasingly high and that the demand for funds in the hog industry is becoming increasingly great, whereas the supply of funds available from the financial markets remains seriously inadequate. Moreover, defects and high risks associated with hog breeding, abnormal fluctuations in market prices, epidemic diseases, natural risks, and other external shocks dramatically affect the hog industry such that a liquidity shortage increases the liquidity risk of firms. The contractual relationship involved with commercial credit causes the negative effects of external shocks acting on other firms in the hog supply chain to generate a systemic risk that contributes to liquidity constraints in the whole hog supply chain.

The impact of external shocks on the health and stability of the hog market and the ability to effectively alleviate the liquidity constraints caused by such external shocks should not be ignored; it is a problem that needs to be solved for all of the nodal enterprises in the hog supply chain. By studying the impact of different degrees of liquidity constraints caused by external shocks on the hog supply chain’s nodal enterprises and ranking the liquidity constraints borne by nodal enterprises, we can gain the ability to alleviate the risk faced by nodal enterprises and help them find the best solutions when they face different degrees of external shocks, thus helping the supply chains maximize their resilience and inhibit the impact of external shocks on nodal enterprises. In the end, this study can help maintain the stability of the hog market and the supply chain, which has practical significance. The liquidity constraints caused by external shocks affect the normal production of hog enterprises, and the financing effect of commercial credit, which can effectively alleviate liquidity constraints, is more pronounced for enterprises–and for small and medium-sized enterprises that are subject to a poor external financial environment in particular [5–7]. Therefore, we built an input-output model based on commercial credit between hog firms and incorporated external shocks into the input-output system, all from the perspective of commercial credit. Using this model, we assessed the liquidity risk of hog firms and the systemic risk of the hog supply chain and rated the external shocks suffered by nodal enterprises in the hog supply chain using the threshold calculation formula of external shocks. The conclusions are as follows. If the external shock is less than a certain value, the current profits of the hog enterprise can entirely make up for the loss caused by external shocks, and the production of the firm will return to its state of equilibrium. With increased external shocks, the liquidity constraints faced by enterprises increase, as do the negative effects on the input and output of the enterprise. They will also affect the input and output of enterprises in the next period and even in many periods after the current period if the enterprises cannot regain the state of equilibrium. If the external shock is large enough, liquidity constraints will seriously restrict the production input of the enterprise, which then leads to a deceleration of production input and may ultimately result in bankruptcy. Furthermore, the external shocks will be transmitted to other firms in the hog supply chain based on commercial credit, which may lead to systemic risk throughout the entire supply chain. In this case, until a strong firm recovers the liquidity shortage through either endogenous or exogenous financing, the spread of the liquidity risk is terminated [2–3] and others have demonstrated the significant role of commercial credit in alleviating liquidity constraints. Moreover, the results show that commercial credit financing (an important short-term financing channel within the supply chain that is extremely important to the financing of numerous small and medium-sized enterprises that are in a weak position in the supply chain) plays an important role in accelerating capital turnover and in lubricating production and circulation in the hog industry. The threshold calculations of external shocks can serve as warnings to the nodal enterprises in the hog supply chain, and the enterprises can analyze their own situation based on the calculations and determine the degree of external shocks they face by using real values in the calculation to understand their level of liquidity constraints and respond appropriately. With the adjustment of the economic structure, stable growth of the new normal of economy (The ‘new normal’ refers to the ‘new normal of the economy’, not a separate noun. The ‘new normal’ of the economy is the symmetry of the economic structure, and the sustainable development of the economy based on the symmetry of the economic structure, including the sustainable and steady growth of the economy), the hog industry has entered into its new normal period with respect to the optimization and upgrading of the industrial structure with the goal of large-scale industrialization. We believe that, as a pillar industry of agriculture in China, the hog industry should vigorously promote the development of supply chain management. the structure of the hog industry supply chain should be constantly adjusted to optimize the industrial upgrading and organizational form of the hog supply chain, and should connect upstream and downstream enterprises with the intent of establishing effective benefit distribution, along with risk- and information-sharing mechanisms and to accelerate horizontal and vertical integration in the chain.

Based on the above conclusions, in this paper, we obtain the following important theoretical research enlightenment based on liquidity constraints and risk, commercial credit, and supply chain financing problems:

1. Under the background of the enterprise competition transferring to the hog supply chain as a whole, participating in competition, it is necessary to further study the overall performance of the hog supply chain under different supply chain financial models and its influencing factors, as well as the alliance formation form and service standards between the financial participants of the “hog-related” supply chain. In order to scientifically measure the liquidity constraints and risk thresholds, enable enterprises to understand the risks they face and find countermeasures. These are important for guiding the steady development of the hog industry.
2. The main participants involved in the relationship of cooperation and competition that are “dabbling in” the hog supply chain are numerous and have different goals. For example, professional logistics providers in fresh agricultural products and commercial banks who enjoy the financing function are important for sustainable development and for attaining the equilibrium value of the best business structure and credit level of every participant in the financial framework of the supply chain.
3. As an important component of hog supply chain finance that is influenced by national macro financial policy, commercial credit is closely related to external macro environment factors, such as financial markets. Studying the impact and mechanism actions of financial policy regarding the agricultural industry (including the hog industry) is of great practical significance to promote the development of supply chain finance, particularly during financial crises or downward economic cycle.

This study establishes the equilibrium state equation and the disequilibrium state equation under external shock are established, based on scale returns remain unchanged and the combination of the variables of production (labor and inventory). Therefore, if we further consider the change of labor elasticity based on the existing research, we will find that under the condition of changes in labor parameters, when the supply chain node enterprises are subject to external shocks, risk threshold for unexpected liquidity restrictions caused by liquidity risk and systemic risk.

## Supporting information

S1 File Annual slaughter of hog of four kinds of scale. (XLSX)

S2 File Table A in S2 File. Medium-scale hog-breeding enterprise profit for 2007–2014. Table B in S2 File. Table of the proportion of various interest rate ranges of RMB loans to financial institutions in 2013. Table C in S2 File. From 2007 to 2014, commercial credit loan interest rate of scale pig breeding was increased.
(XLS)
